# Social media utilization, mindfulness practice, and psychological distress of nonprofit workers in China: mediation effects of positive and negative affect

**DOI:** 10.3389/fpubh.2025.1405372

**Published:** 2025-04-02

**Authors:** Qianyi Tu, Chienchung Huang, Bin Tu

**Affiliations:** ^1^School of Public Policy and Management, Guangxi University, Nanning, China; ^2^School of Social Work, Rutgers, The State University of New Jersey, New Brunswick, NJ, United States; ^3^School of Public Administration, Guangdong University of Foreign Studies, Guangzhou, China

**Keywords:** mindfulness practice, negative affect, positive affect, psychological distress, social media utilization

## Abstract

**Introduction:**

In the contemporary landscape, the intersection of technology and human behavior has given rise to transformative trends, prominently featuring the emergence of social media. Nevertheless, studies show that adults who heavily depend on these platforms may affect their psychological distress. Conversely, a growing body of research indicates that engaging in mindfulness practices can regulate emotional reactions and contribute to enhanced mental health, resulting in a reduction of psychological distress.

**Methods:**

In this investigation, a cross-sectional survey involving 318 adult nonprofit employees in China was conducted to explore the impact of social media utilization and mindfulness practice on psychological distress. The study specifically aimed to examine whether positive and negative affect mediate the relationships between these variables. Structural Equation Modeling was employed to analyze both the direct and indirect effects of social media utilization and mindfulness practice on psychological distress through positive affect (PA) and negative affect (NA).

**Results:**

Social media utilization positively influenced positive affect (PA) (*β* = 0.15, *p* < 0.01) but showed no significant impact on negative affect (NA). Mindfulness practice displayed a positive effect on PA (*β* = 0.30, *p* < 0.001) and a negative effect on NA (*β* = −0.10, *p* < 0.10). PA exhibited a negative effect on psychological distress (*β* = −0.12, *p* < 0.01), whereas NA had a positive effect on psychological distress (*β* = 0.75, *p* < 0.001). The total effect of mindfulness practice on psychological distress was −0.11 (*p* < 0.01), while social media utilization did not have a significant effect on distress.

**Discussions:**

The results indicated that participating in mindfulness practice boosts PA and simultaneously diminishes NA and psychological distress. Despite social media utilization being linked to heightened PA, it did not demonstrate significant effects in mitigating NA or psychological distress. This study underscores the significance of advocating for mindfulness practice as a public health strategy to alleviate NA and psychological distress, while also fostering PA among adults in China.

## Introduction

In the modern landscape of the world, the intersection of technology and human behavior has given rise to a myriad of transformative trends. Among these, the widespread utilization of social media stands out as a significant phenomenon, exerting a profound influence on various aspects of individuals’ lives. Though the definition of social media is myriad, it can be defined as “Internet-based channels that allow users to opportunistically interact and selectively self-present, either in real-time or asynchronously, with both broad and narrow audiences who derive value from user-generated content and the perception of interaction with others (1). Although social media is primarily linked to personal communication and networking, a growing body of research suggests that adults increasingly relying on these digital platforms may impact their psychological well-being (2–5, 51). Studies have uncovered associations between internet and social network use and a decrease in psychological distress over time (3, 4, 6). Conversely, other research indicates that excessive social media use or addiction is positively associated with psychological distress (2, 7, 8).

Mindfulness is a mental state marked by a non-judgmental and non-reactive awareness of the current moment, displaying both trait-like characteristics and variability among individuals and over time (9, 10). Mindfulness practice refers to engaging in activities or exercises aimed at nurturing mindfulness. This includes formal practices like meditation, as well as informal ones where mindfulness skills are applied to everyday activities, such as washing dishes or strolling through the park with awareness (11, 12). Empirical studies indicate that engaging in mindfulness practices is linked to a multitude of favorable outcomes, encompassing social and emotional competence, overall health, and well-being (13–15). Earlier studies also propose that mindfulness can effectively regulate stress reactions [e.g., (11, 16, 17)], with a growing body of evidence suggesting that incorporating mindfulness into one’s routine can lead to stress reduction and enhanced mental well-being. These positive effects, in turn, are associated with lower levels of burnout and psychological distress (18–21).

Psychological distress manifests as a state of emotional anguish marked by stress-related events, significantly impeding daily life (22, 23). Typically, this distress is characterized by symptoms of depression or other somatic complaints, elevating the risk of various behavioral disorders and illnesses, including mood disorders (24, 25) and even suicidal behavior (26, 27).

Considering the substantial impact of psychological distress on an individual’s mental and behavioral well-being, it is imperative to explore the potential influences of social media use and mindfulness practice on such distress. Additionally, investigating whether positive and negative affect mediate these relationships is crucial. Positive affect (PA) and negative affect (NA) are important elements of well-being (28). PA encompasses positive moods or emotions such as optimism, while NA involves negative moods or emotions such as anxiety (29, 30). This study aims to scrutinize the connections among social media utilization, mindfulness practice, and psychological stress within a sample of adult employees in China. By delving into the mechanisms through which these platforms act as tools for reducing psychological distress, the article seeks to contribute to the evolving understanding of how social media and mindfulness practices impact PA, NA, and psychological distress in China. The findings hold implications for individual well-being and offer insights into potential mechanisms for alleviating psychological distress.

The conceptual framework of this study draws upon the transactional model of stress and coping (31) and the broaden-and-build theory (32). The transactional model views stress as a dynamic interplay between individuals and their environment, where individuals assess available resources to cope with stressors. This research examines social media usage and mindfulness practices as potential coping resources within this model. The coping strategies adopted are expected to influence individuals’ well-being, measured by PA and NA in this study. Fredrickson’s broaden-and-build theory suggests that PA can expand individuals’ cognitive and behavioral repertoires, enabling them to accumulate essential physical, psychological, and social resources instrumental in achieving positive accomplishments and coping with mental health challenges (32–34). Conversely, NA reduces thought-action repertoire, hindering people from obtaining necessary resources for performance improvement and health, thereby increasing the risk of negative physical and mental health outcomes (29, 35). Thus, within this framework, social media use and mindfulness practices are viewed as integral components of coping strategies individuals employ to navigate daily challenges, ultimately impacting their PA, NA, and psychological distress.

## Methods

### Data and sample

This study gathered data through an anonymous web-based survey targeting adult employees in the nonprofit sector in Guangdong, China. The inclusion criteria encompassed adult employees aged 18 years and older, employed within registered nonprofit organizations located in Guangdong. The study aimed to include a total of 100 nonprofit organizations. Employing a stratified sampling approach, the sample size for nonprofit organizations in each of the 21 cities was determined based on their respective proportions within Guangdong. Subsequently, nonprofit organizations were randomly selected within each city for inclusion in the study. Within each selected organization, four employees were randomly chosen for participation, resulting in a target sample size of 400 individuals. In collaboration with the Guangdong Social Organizations Federation, invitations to participate in the online survey were extended to employees of these nonprofit organizations in November 2021, with reminders sent out 14 days after the initial invitations. By December 31, 2021, we had received a total of 318 responses. Before conducting the survey, an informed consent process was implemented, explicitly conveying to participants the voluntary nature of their involvement and their right to discontinue the survey at any point. The survey, facilitated through the Questionnaire Star platform, was structured to mandate respondents’ completion of all questions for online submission. Consequently, there were no missing data or incomplete surveys among the collected data. On average, respondents spent approximately 16 min completing the questionnaire. The research protocol obtained approval from the research review committee at one of the co-authors’ universities in China. The sample comprised about 71% females, with an average age of 33, ranging from 21 to 58. A majority of participants had achieved a college degree (57%) or higher education (5%), while 38% had attained high school education or below. Approximately 59% of the sample were married at the time of the survey. The mean length of employment was 5.5 years. The demographics of sample, including high percentage of females, align with findings in previous studies in Chinese nonprofit workforce (36, 37).

### Measures

The assessment of psychological distress utilized the Kessler 6 Psychological Distress Scale (“K6”) (25, 38–40). The K6’s measurement of psychological distress has been previously tested and demonstrated reliability, with a Cronbach’s alpha value of 0.89 in Kessler et al. (38). Participants were required to self-report the frequency of their experience of psychological distress over the past 30 days. Responses were rated on a 5-point scale, ranging from 0 (“none of the time”) to 4 (“all of the time”). The sum of all responses for each participant resulted in a psychological distress score within the range of 0–24. In this study, the K6 scale demonstrated a Cronbach’s alpha value of 0.90.

Positive affect and negative affect (PANA) were evaluated using the 10-item short-form version of the International Positive and Negative Affect Schedule [I-PANAS-SF; (41)]. This instrument has demonstrated good internal reliability and convergent and criterion-related validity. For example, the Cronbach’s alpha values were 0.78 and 0.76 for PA and NA in Thompson study (41). The I-PANAS-SF prompts respondents to indicate the frequency with which they experienced various emotions (e.g., inspired, determined, hostile, and upset) in the past 2 weeks. Response options range from 1, “never,” to 5, “always.” Scores were averaged for items corresponding to positive affect (PA) and negative affect (NA), with possible scores ranging from 1 to 5. In this study, the PA and NA scales exhibited a Cronbach’s alpha value of 0.76 and 0.84, respectively.

Mindfulness practice was assessed by inquiring whether respondents engaged in specific activities, including formal practices (e.g., meditation) and informal practices (e.g., mindful nature observation, such as walking in the park mindfully) (21). Participants rated their practice on a 7-point Likert scale: 0 (never), 1 (once or less every 2 months), 2 (once per month), 3 (2 or 3 times per month), 4 (once per week), 5 (2 or 3 times per week), and 6 (once or more per day).

Social media utilization was gaged by inquiring about the frequency of respondents’ use of social media on a 7-point Likert scale: 0 (never), 1 (once or less every 2 months), 2 (once per month), 3 (2 or 3 times per month), 4 (once per week), 5 (2 or 3 times per week), and 6 (once or more per day).

### Analytical approach

We conducted descriptive analyses to initially explore the sample characteristics of key variables. Subsequently, Pearson’s correlation analysis was employed to investigate the relationships among these variables. Structural Equation Modeling (SEM) was then carried out to simultaneously examine the direct and indirect effects of social media utilization and mindfulness practice on psychological distress through PA and NA. The Maximum Likelihood estimation method was utilized in SEM. Model-to-data fit was evaluated using several fit indices, including Chi-square statistics, Comparative Fit Index (CFI), Standardized Root Mean Square Residual (SRMR), and Root Mean Square Error of Approximation (RMSEA). A good model-to-data fit was indicated by values of Chi-square statistics >0.05, CFI > 0.95, SRMR <0.08, and RMSEA values <0.08. Additionally, we performed regression analyses with confounding variables, such as gender, age, and education characteristics. The regression results regarding key variables, available upon request, did not exhibit significant deviations from those reported in this study. All analyses were conducted using STATA statistical software, version 16.0.

## Results

Table 1 presents the descriptive statistics for the variables. On average, the sample reported a social media utilization score of 5.0 (SD = 1.6) and a mindfulness practice score of 3.1 (SD = 1.8). In our sample, the mean scores for PA, NA, and psychological distress were 3.3 (SD = 0.8), 1.9 (SD = 0.8), and 4.4 (SD = 4.5), respectively. The findings indicate that the nonprofit employees in our sample frequently used social media, approximately 2 or 3 times per week, while engaging in mindfulness practice less often, around once a week. Although most employees did not report high psychological distress and demonstrated modest levels of PA and relatively low NA, some individuals in the sample experienced elevated distress, low PA, and high NA.

**Table 1:** Descriptive statistics and correlations of key variables.

Variables	Mean (S.D.)	1	2	3	4	5
1. Social media utilization	5.0 (1.6)	—				
2. Mindfulness practice	3.1 (1.8)	0.20 ***	—			
3. Positive affect	3.3 (0.8)	0.21 ***	0.33 ***	—		
4. Negative affect	1.9 (0.8)	−0.09	−0.11 *	0.07	—	
5. Psychological distress	4.4 (4.5)	−0.04	−0.13 *	−0.07	0.74 ***	—

*n* = 318. * *p* < 0.05; ** *p* < 0.01; *** *p* < 0.001.

Results from the correlation analysis indicate a positive correlation between social media utilization and mindfulness practice (*r* = 0.20, *p* < 0.001), as well as a positive correlation between social media utilization and PA (*r* = 0.21, *p* < 0.001). However, social media utilization did not exhibit a significant correlation with NA or psychological distress in our sample. Mindfulness practice showed a positive correlation with positive affect (*r* = 0.33, *p* < 0.001) and a negative correlation with both negative affect (*r* = −0.11, *p* < 0.05) and psychological distress (*r* = −0.13, *p* < 0.05). Positive affect did not display correlations with negative affect or psychological distress, while negative affect exhibited a positive correlation with psychological distress (*r* = 0.74, *p* < 0.01).

**Figure 1:**
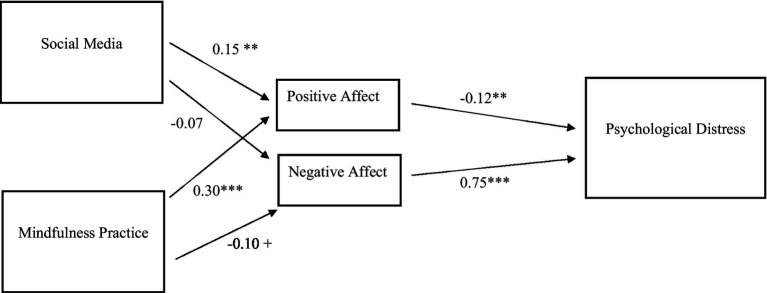
Standardized estimates of social media utilization, mindfulness practice, PANA, and Psychological Distress. *N* = 318; + *p* < 0.10, ** *p* < 0.01, ***p < 0.001.

Figure 1 illustrates the standardized coefficients derived from the Structural Equation Model (SEM) results. The recommended model fit well with the data: *χ*2 (2) = 2.2, *p* > 0.05, CFI = 0.99, RMSEA = 0.02, SRMR = 0.01. The findings indicate that the model fits the data well and that PA and NA fully mediated the effects of social media utilization and mindfulness practice on psychological distress. Specifically, social media utilization had a positive effect on PA (*β* = 0.15, *p* < 0.01) but no significant effect on NA. Mindfulness practice exhibited a positive effect on PA (*β* = 0.30, *p* < 0.001) and a negative effect on NA (*β* = −0.10, *p* < 0.10). PA, in turn, demonstrated a negative effect on psychological distress (*β* = −0.12, *p* < 0.01), while NA had a positive effect on psychological distress (*β* = 0.75, *p* < 0.001). Social media utilization had an indirect effect of −0.07 on psychological distress through PANA (*p* < 0.10) while the indirect effect of mindfulness on psychological distress through PANA was −0.11 (*p* < 0.01). Finally, the total effects of both social media utilization and mindfulness practice on psychological distress were identical to their corresponding indirect estimates, as positive and negative affect (PANA) fully mediated their impacts on psychological distress.

## Discussion

The descriptive results suggest that adult employees in the nonprofit sector in China generally exhibit good mental health, characterized by high PA and relative low NA and psychological distress. Moreover, social media utilization appears moderate, occurring 2–3 times per week, while the extent of mindfulness practice is moderately low on average, around once a week. The SEM findings pointed out that social media usage was found to have a positive impact on PA but did not show a significant effect on NA. On the other hand, mindfulness practice exhibited a positive effect on PA and a negative effect on NA. PA was negatively associated with psychological distress, while NA had a positive association with psychological distress. Mindfulness practice appears to reduce psychological distress via its effects on PA and NA, while social media usage did not significantly influence the distress.

The positive relationship between social media use and PA aligns with prior research indicating that moderate engagement with social media can enhance well-being by fostering social connections, providing emotional support, and facilitating access to beneficial information (3, 4, 6, 51). However, the lack of a significant effect on NA and psychological distress suggests that social media usage may not necessarily serve as a protective factor against negative emotional states. This finding is particularly relevant given the mixed evidence in the literature, where some studies highlight the potential for excessive social media use to contribute to psychological distress (2, 7, 8). The present study does not differentiate between active versus passive engagement or assess the intensity of usage, which are critical factors that may influence mental health outcomes. Future research should incorporate measures of social media engagement types and screen time to determine whether more intensive use is linked to heightened distress.

The adult participants in our study demonstrated relatively low engagement in mindfulness practice, averaging about once per week, encompassing both formal meditation and mindful nature observation activities, such as walking in the park mindfully. However, despite its infrequent occurrence, mindfulness practice exhibited stronger effects on mental health compared to social media utilization. This practice notably increased PA and reduced NA and psychological distress. These findings align with prior research highlighting the positive impact of mindfulness practice on mental health (11, 20, 21, 42). Given the low prevalence of mindfulness practice and its substantial positive effects on mental health, employers in the Chinese nonprofit sector may contemplate introducing mindfulness practice training for their employees. Encouraging employees to engage in mindfulness, both formally and informally, could yield significant benefits in terms of improving work productivity and fostering holistic employee well-being (12, 43).

The findings of this study carry both practical and theoretical implications that can inform interventions and further research in the field of social media use and mindfulness practice within the Chinese nonprofit sector and beyond. First, for practical implications, employers within the Chinese nonprofit sector can leverage the findings to design tailored interventions aimed at promoting employee well-being (43). Implementing mindfulness practice programs can be particularly beneficial, given it demonstrated positive effect on PA and negative effects on NA and psychological distress (11, 12). Secondly, organizations can incorporate mindfulness practices into existing employee support programs, offering resources and guidance for both formal meditation practices and informal mindful activities (44). By encouraging regular engagement in mindfulness, employers can foster a culture of well-being and resilience within the workplace (12, 45). Thirdly, insights from this study can also inform policy development aimed at promoting mental health awareness and support within the nonprofit sector and beyond. Policies that prioritize mental health resources and training programs, including mindfulness practices, can contribute to creating healthier work environments and enhancing overall employee satisfaction and productivity (46).

With respect to theoretical implications, this study underscores the importance of considering cultural context in understanding coping strategies and mental health outcomes. It highlights the unique dynamics within the Chinese nonprofit sector and emphasizes the need for culturally sensitive approaches in mental health research and interventions. In addition, the differential effects of social media usage and mindfulness practice on mental health outcomes contribute to the development of the transactional model of stress and coping and the broaden-and-build theory (31, 32). Integrating both social media usage and mindfulness practice factors as coping strategies can enrich theoretical frameworks aimed at understanding and promoting mental well-being. Social media platforms often provide immediate gratification and passive consumption of content, which may offer distraction but does not necessarily promote active engagement or self-reflection (47, 48). In contrast, mindfulness practice involves cultivating present-moment awareness and active engagement with one’s internal experiences, promoting self-awareness, emotional regulation, and acceptance (49, 50). Finally, while not explicitly explored in this study, the gender-specific implications of coping strategies and mental health outcomes warrant further investigation. Future research can delve into gender differences in social media usage, mindfulness practice, and their impacts on mental health within the Chinese nonprofit sector, contributing to a more nuanced understanding of gender dynamics in mental health.

This study has several limitations. Firstly, as cross-sectional data were employed in our analyses, we can only infer associative relationships among social media utilization, mindfulness practice, positive and negative affect (PANA), and psychological distress, rather than establishing causal connections. As a future research agenda, conducting a longitudinal or experimental study would help strengthen the conclusions by allowing for a better understanding of causal relationships among social media utilization, mindfulness practice, PANA, and psychological distress, rather than relying solely on associative inferences from cross-sectional data. Secondly, there are likely unobserved variables that may have influenced our estimates.

For example, while this study measures the frequency of social media use, it does not differentiate between types of engagement, such as passive (e.g., scrolling or viewing content) versus active (e.g., posting, commenting, or interacting). This distinction is important, as previous research suggests that different types of social media use may have varying effects on psychological outcomes. Future studies should consider incorporating measures that capture these nuances to provide a more comprehensive understanding of the relationship between social media use and mental health. Another limitation is the reliance on self-reported data from participants, which may introduce reporting errors. Fourthly, while our measure of mindfulness practice encompassed both formal and informal ones, it’s worth noting that the feasibility of engaging in these practices could vary depending on geographic location. For example, individuals in rural areas might find it easier to engage in mindful activities like walking in nature compared to those in urban settings. Conversely, individuals in urban areas might have easier access to meditation facilities compared to those in rural settings. Further investigation into the accessibility and frequency of mindfulness practices across regions is needed to fully understand these dynamics. Lastly, it’s crucial to acknowledge the limitation of external validity in our study. Our sample was drawn adult nonprofit workers in a single province of China, Guangdong. Consequently, the generalizability of our findings to other adult workers in Guangdong or nonprofit workers in different provinces across China is constrained.

## Conclusion

This study analyzed data from 318 nonprofit employees in China to examine the influence of social media utilization and mindfulness practice on psychological distress, with a focus on the mediating role of PA and NA. Our findings contribute to the growing body of cross-cultural research on mindfulness, demonstrating that mindfulness practice not only enhances PA but also effectively reduces NA and psychological distress. These results reinforce the role of mindfulness as a valuable mental health strategy, particularly in professional settings where stress management is crucial. Although social media utilization was associated with increased PA, it did not significantly impact NA or psychological distress. This suggests that while moderate social media engagement may contribute to positive emotions, it does not necessarily buffer against negative affect or distress. Given prior literature indicating that excessive or passive social media use may have adverse mental health effects, future research should further explore the intensity and type of social media engagement to clarify its broader implications. Overall, this study underscores the importance of promoting mindfulness practice as a meaningful intervention for enhancing emotional well-being and reducing distress among nonprofit employees in China. Encouraging more frequent mindfulness engagement may provide a practical and effective approach to fostering mental resilience in professional and social contexts. Future research should consider longitudinal and experimental designs to establish causal relationships and further investigate the complex dynamics between social media use, mindfulness, and mental health outcomes.

## Data Availability

The raw data supporting the conclusions of this article will be made available by the authors, without undue reservation.
